# Observation of Interactions in Adolescent Group Therapy: A Mixed Methods Study

**DOI:** 10.3389/fpsyg.2017.01188

**Published:** 2017-07-24

**Authors:** Eulàlia Arias-Pujol, M. Teresa Anguera

**Affiliations:** ^1^FPCEE Blanquerna, Ramon Llull University Barcelona, Spain; ^2^Faculty of Psychology, University of Barcelona Barcelona, Spain

**Keywords:** group therapy, adolescent interactions, mixed-method, polar coordinates analysis, mentalization

## Abstract

Group psychotherapy is a useful clinical practice for adolescents with mental health issues. Groups typically consist of young people of similar ages but with different personalities, and this results in a complex communication network. The goal of group psychoanalytic psychotherapy is to improve participants' mentalization abilities, facilitating interactions between peers and their therapist in a safe, containing environment. The main aim of this study was to analyze conversation turn-taking between a lead therapist, a co-therapist, and six adolescents over the course of 24 treatment sessions divided into four blocks over 8 months. We employed a mixed-methods design based on systematic observation, which we consider to be a mixed method itself, as the qualitative data collected in the initial observation phase is transformed into quantitative data and subsequently interpreted qualitatively with the aid of clinical vignettes. The observational methodology design was nomothetic, follow-up, and multidimensional. The choice of methodology is justified as we used an *ad*-*hoc* observation instrument combining a field format and a category system. Interobserver agreement was analyzed quantitatively by Cohen's kappa using the free QSEQ5 software program. Once we had confirmed the reliability of the data, these were analyzed by polar coordinate analysis, which is a powerful data reduction technique that provides a vector representation of relationships between categories. The results show significant relationships between the therapist and (1) the activation of turn-taking by the participants and the co-therapist and silence and (2) conversation-facilitating interventions and interventions designed to improve mentalization abilities. Detailed analysis of questions demonstrating interest in others showed how the communication changed from radial interactions stemming from the therapist at the beginning of therapy to circular interactions half way through. Repetition was found to be a powerful conversation facilitator. The results also illustrate the role of the therapist, who (1) did not facilitate interventions by all participants equally, (2) encouraged turn-taking from more inhibited members of the group, (3) stimulated conversation from the early stages of therapy, and (4) favored mentalization toward the end. Despite its complexity, polar coordinate analysis produces easy-to-interpret results in the form of vector maps.

## Introduction

Peer groups are a natural setting for young people (Erikson, 1968). In the social context, Malekoff (2014) and Tellerman (2001) consider group work to be a protective factor for teenagers, pre-teenagers, and their families. In the field of public health, group psychotherapy is a useful clinical practice for adolescents with varying mental health issues (Reid and Kolvin, 1993; Cramer-Azima, 2002). Adolescent mental health disorders have increased over the last three decades (Nuffield Foundation, 2013) and today's teenagers have higher rates of anxiety, behavioral problems, and mood disorders (Merikangas et al., 2010).

Little has been published on group therapy in children or adolescents. Most of the studies conducted to date have reported on brief cognitive-behavioral interventions with specified diagnostic populations (Pollock and Kymissis, 2001). There has also been research into group counseling and psychotherapy with children and adolescents that indicates that the peer feedback that occurs in such settings is a key part of the process of change (Shechtman, 2007). The theoretical orientation behind this study was a combination of interpersonal and psychodynamic theories. Pingitore (2016) validated the benefits of interpersonal group therapy, an approach originally proposed by Yalom (2005), by quantitatively analyzing audio recordings of interventions by eight adolescents who took part in a process-oriented psychotherapy group for 3 months. Within a Kleinian psychoanalytic framework and following the contributions of Devi and Fenn (2012) published a systematic thematic analysis of a latency-aged children's group. Through clinical extracts, they showed how the children shifted from paranoid-schizoid functioning to depressive functioning over the course of therapy. They concluded that psychotherapy was beneficial in latency-aged children, as it provided them with the opportunity to observe and try to attach meaning to the interactions of other people, to respond to these interactions, to initiate contact and to help and be helped in a safe environment. Such experiences improve individuals' ability to recognize and observe mental states in both themselves and others and to develop empathy.

More research has been conducted in adults. A recent meta-analysis of group psychotherapy for social anxiety disorders concluded that group interventions were as effective as individual psychotherapy or pharmacotherapy (Barkowski et al., 2016). Group therapy is also beneficial for adults with moderate or severe depression (Pylvänäinen et al., 2015) or eating disorders (Simpson et al., 2010) and it has been shown to reduce symptoms of anxiety, depression, and avoidance in adults with personality disorders (Skewes et al., 2015). Schwartze et al. (2016) recently published a meta-analysis that showed that cognitive behavioral therapy was effective for patients with obsessive-compulsive disorder. Another randomized controlled study that compared the outcomes of short- and long-term psychodynamic psychotherapy (90-min weekly sessions for 20 or 80 weeks) in 167 adult outpatients with mood, anxiety, and personality disorders found that patients in both groups made significant gains, and concluded that short- and long-term therapy seemed equally effective for typical outpatients seeking group psychotherapy, with the exception of symptomatic distress, for which a more favorable treatment effect was found for the long-term therapy (Lorentzen et al., 2013). A recent open prospective controlled study showed the efficacy of short-term dynamic group psychotherapy (37–39 sessions lasting 75 min over 9 months) in primary care patients with depressive symptoms (Bros et al., 2016).

In pyschotherapy research, there is growing concern for integrating qualitative methods, which provide a more holistic view of the person, and quantitative methods, which seek to provide a more objective view (Lutz and Hill, 2009). Despite the dearth of publications in the last decade, there are encouraging signs of a growing interest in the use of mixed-methods research in psychology (Roberts and Povee, 2014). By integrating complementary perspectives derived from quantitative and qualitative methods and analyses, mixed-methods research offers both rigor and flexibility and is likely to see an increase in future years (Anguera and Hernández-Mendo, 2016).

In this article, we describe the results of a study based on systematic observation, which we consider to be a mixed method in itself (Anguera and Hernández-Mendo, 2016). The study consisted of systematically observing video-recordings of adolescent group therapy sessions over a period of several months. The observation produced a large set of qualitative conversational data, subsequently analyzed quantitatively via polar coordinate analysis to detect changes in behaviors over the course of therapy.

The aim of the group therapy analyzed was to promote autonomy and maturity through interactions between peers and their therapist in a safe, containing environment (Torras de Beà, 2013). Group sessions of this type produce complex communication networks. Participants are typically young people of similar ages with different personalities who have difficulty relating to others and often perform poorly at school.

Psychodynamic interventions have been described as “conversation therapies,” as the relationship between the person seeking treatment and the therapist forms the basis of the therapy (Malmberg and Fenton, 2008). We studied group communication as a conversation in which we analyzed turn-taking (who) and content (what).

Foulkes (1986) described two roles for group analysis leaders, or conductors: a role as dynamic-administrator and a role as analyst-interpreter. The function of the first is to set up the group, establish norms and boundaries, and create a safe, supportive, and containing environment designed to increase participation, expressiveness, and interaction and communication. The function of the second, by contrast, is related to mental activity, and consists of observation, listening, and understanding, and the ability to put into words everything they are understanding.

In the group studied, interventions by a therapist largely seek to (a) facilitate conversation and (b) promote mentalization, i.e., stimulate thought, reflection, and understanding about oneself and one's relationships with others.

In the *ad*-*hoc* observation instrument used in the study, we labeled this first group of interventions DYN, as they have a dynamic, stimulating function. They are interventions in the form os a request or question in which the emitter (the therapist or participants) show interest in the life of the receiver. Demonstrating interest in others by asking questions, allowing them to intervene, and showing curiosity in their answers is considered to be a specific benefit of group therapy as opposed to individual therapy (Yalom, 2005; Torras de Beà, 2013). In previous studies, we saw that DYN interventions were very common in all sessions and that over the course of therapy, their use increased among participants and decreased among therapists, forming significant behavioral patterns (Arias and Anguera, 2004, 2005; Arias, 2011).

The second group of interventions in the observation instrument was called MNT to reflect the concept of mentalization described by Fonagy et al. (Fonagy, 1991; Fonagy et al., 1995), which is understood as the ability to explain and give meaning to one's own behaviors and those of others within a process of mental representation, thoughts, desires, and expectations. This ability is not innate: it needs to be developed within a safe, affective environment, which in psychoanalytic group psychotherapy is achieved by maintaining a stable internal and external setting while containing anxieties. MNT interventions are part of the therapist's role (Bateman and Fonagy, 2012), while DYN interventions correspond to either the therapist or the participants over the course of the sessions.

At the beginning of these group sessions, communication is generally radial, i.e., it diverges outwards toward the participants from the formal leader of the group, the therapist. With time, it becomes circular, with participants spontaneously intervening and demonstrating interest in each other. This shift in the direction of communication is an indicator of the group process, and our aim was to objectively analyze this process by studying the therapist's interventions.

The main aim of this study was to apply polar coordinate analysis to analyze conversation turn-taking and DYN and MNT interventions in a group therapy program involving a lead therapist, a co-therapist, and six adolescents. The program consisted of 24 group sessions, divided into four blocks, held over a period of 8 months.

## Materials and methods

### Design

In this mixed-methods study, we applied systematic observation, which meets the rigorous standards of scientific inquiry while at the same time offers the flexibility needed in real-life settings. Observational methodology permits the capture of spontaneous behaviors as they occur in a natural environment (Sackett, 1978; Anguera, 1979, 2003; Bakeman and Quera, 1995b, 2011; Portell et al., 2015a,b). It is thus an ideal methodology for studying communication in group therapy, and has proven to be suitable for studying the changes that occur over the course of therapy (Pascual-Leone et al., 2009).

There are eight possible study designs in observational methodology (Blanco-Villaseñor et al., 2003; Sánchez-Algarra and Anguera, 2013). The design used in this study was N/F/M (nomothetic/follow-up/multidimensional). It was *nomothetic* because we conducted a parallel analysis of the therapist, the co-therapist, and six adolecents, *follow-up* because we performed both intersessional analyses (24 successive sessions) and intrasessional analyses (sequential recording of all behaviors from the start to finish of each session), and multidimensional because the *ad*-*hoc* observation instrument contained various dimensions selected on the basis of the theoretical framework and our experience.

The systematic observation was non-participative and the behaviors were highly perceivable.

### Participants

There were eight participants: the therapist (T), the co-therapist (coT), and six adolescents (G, D, JM, F, L, M). The adolescents (four boys and two girls) had requested support at the Center for Child and Adolescent Mental Health of the *Eulàlia Torras de Beà* Foundation in Barcelona, Spain. They all had difficulties relating to others and difficulties learning at school; they had normal or normal-low intelligence according to the Weschler Intelligence Scale for Children–Fourth Edition (WISC-IV, Weschler, 2006). Two had a mild behavioral disorder, three had anxiety problems, and one tended to disconnect (Table 1, codes ICD-9-CM, Ministerio de Sanidad, Servicios Sociales e Igualdad, 2014).

**Table 1:** Patient characteristics.

**Pseudonym**	**Age**	**Gender**	**Total IQ**	**Diagnosis (ICD-9-CM CODES)**
Gabriel	14	Male	111	313.83/315.02/313.81.1
Danny	14	Male	110	309.23/313.0
John M.	14	Male	92	309.23/300.00.1/301.4.01
Fred	13	Male	90	309.23/297.3
Lucy	15	Female	84	309.23/315.5/313.81.1
Meg	13	Female	110	309.23/300.21/300.2

*Pseudonyms have been used to protect confidentiality*.

The inclusion criteria were (a) an age of 12–15 years and (b) recommendation for group therapy following diagnostic evaluation at the Mental Health Center. The exclusion criteria were (a) anticipated difficulty attending all the therapy sessions and (b) contraindication for group therapy.

The group was led by an expert therapist, assisted by a co-therapist who participated as an observer. Both were clinical psychologists trained in group psychoanalytic psychotherapy.

In accordance with the principles of the Declaration of Helsinki and the Ethical Code of the General Council of the Official College of Psychologists of Spain, the participants were informed that they were being filmed. They were shown the location of the video cameras, which were positioned discretely to minimize reactivity bias. Informed consent was also obtained from the parents of the minors.

### Instruments

In systematic observation (Anguera, 2003; Sánchez-Algarra and Anguera, 2013), a distinction is made between recording instruments (i.e., those used to record or code data) and observation instruments (purposed-designed instruments to analyze a given subject).

#### Recording instrument

The group sessions were recorded using two video cameras, two microphones, two video units, and two screens comprising a closed-circuit television system. The dataset was built in the software program GSEQ5, v.5.1 (Bakeman and Quera, 2011) using an initial transcription of the video content. In accordance with the principles of the Declaration of Helsinki and the Ethical Code of the General Council of the Official College of Psychologists of Spain, the participants were informed that they were being filmed. They were shown the location of the video cameras, which were positioned discretely to minimize reactivity bias.

According to the terminology proposed by Bakeman (1978), the data recorded were type II data, i.e., they were concurrent (as we considered various dimensions and each behavior needs to be coded using a specific code) and event-based (as the behaviors were coded as they occurred, thereby providing information on order and sequence, two essential factors for our study). It is also possible to record duration, but this was not relevant to the purpose of our study. Once annotated, each behavior generates a co-occurrence of codes (corresponding to the different dimensions) and is methodologcally considered to be a multievent (Bakeman, 1978). A total of 30,436 multievents were coded in our study.

#### Observation instrument

The *ad*-*hoc* observation instrument used in the study combined a field format and category systems. It is a flexible instrument in which the different dimensions considered can be broken down into different categories according to the theoretical framework and experience. Considering the specific goals of the study and based on previous experiences (Arias and Anguera, 2004, 2005), the observation instrument was redesigned to include 15 forms of communication. These forms, or dimensions of communication, were derived from the work of Torras de Beà (2013) on group psychotherapy and of Tusón (1995) and Calsamiglia and Tusón (1999) on conversation analysis.

The 15 dimensions included in the observation instrument are Facilitating of conversation, Reflective function, Expressivity, Defensive expressions, Dislike, Ordering, Humor, Confrontation, Exclamation, Degradation of vocal behavior, Whispering, Touching, Noise, Surrounding noise, and Silence (Table 2). Each of these dimensions was broken down to build a category system that fulfilled the requirements of exhaustivity and mutual exclusivity (Anguera, 2003).

**Table 2:** Dimensions and category systems in the observation instrument for therapists and patients.

**Dimensions**	**Category systems**	**Description**
DYN Facilitating of conversation	DYN = {FF, FO, RP, RT, QA, QC, QV}	*Facilitating of conversation*. Suitable questions or requests to start or enhance dialogue; routines such as greetings and other conversational rituals; requests for clarification; verification questions; full or partial repetitions of a previous intervention in the form of a statement or a question; vocalizations indicating that the communication channel is still open. FF = Phatic function. Vocalization indicating that the communication channel is still open. It indicates continued attention and cooperation, without the addition of new information. Typical vocalizations are “hmmn,” “hum,” or “aha.” FO = Conversational routines or rituals, such as greetings or expressions of gratitude. RP = Total or partial reproduction of a previous utterance in the form of a statement not a question. This could be an answer to a request for clarification or it could have a phatic function, such as, for example, when the speaker simply echoes what a person has just said, indirectly encouraging them to continue. RT = Bringing back a topic of conversation. Intervention in which a participant brings back a subject previously brought up by another participant after a change of subject (CT) or interruption, thus making sure it is not forgotten. QA = Expressive question. Request, question, or series of adequate questions to start or promote dialogue and keep the main topic of conversation flowing. The person gives the turn to another person and shows interest in them. QC = Clarifying question. Question asking for clarification about what is happening. The person intervenes to clarify their own confusion and/or surprise in the form of a question. The speaker asks about a particular topic, doubt, or puzzlement, or about expressions, gestures, noises, or laughter he/she has not understood. It is a strategy used by the therapist when the adolescents are “doing their own thing.” QV = Repetition of a previous statement in the form of a question. It is used to confirm what has just been said. It has a phatic function, as the speaker is conveying that the communication channel is still open. It can also be a strategy to emphasize a particular word or intervention.
MNT	MNT = {MNT}	*Reflective function*. Interventions focused on promoting thought, reflection, and understanding of oneself and one's relationships with others. They seek to stimulate the ability to understand what is happening in the minds of others. They are used by the therapist and can be directed at an individual or at the group as a whole. They include emphatic interventions, which put words to other participants' feelings.
EXP	EXP = {RA EC CD RB}	*Expressivity*. Interventions and answers manifesting the thoughts and/or feelings of the person speaking, adding new content to the conversation; short answers; sequences of words that continue the main subject of conversation; interventions that revisit subjects already dealt with.
DEF	DEF = {RD_N_P CT}	*Defensive expressions*. Interventions in which the participant avoids answering a previous question; verbalizations expressing the opposite of what has been said or done; projection of conflicts onto others; changing of subject.
DIS	DIS = {ED PD}	*Dislike*. Interventions expressing dislike, disagreement, distaste, or defiance.
ORD	ORD = {ORD}	*Ordering*. Prescriptive verbalizations, authoritarian demands (including exclamations).
HUM	HUM = {R EO}	*Humor*. Interventions with a clearly ironic/wry intention, jokes, jibes, and laughter.
CFR	CFR = {CFR}	*Confrontation*. Verbal interventions used by participants to express what they feel is happening in the group or see in some of their peers. They mirror the behavior of another. Peer feedback.
EX	EX = {EX}	*Exclamation*. Onomatopoeic word or words indicating a strong emotion of surprise, joy, or sadness.
S4	S4	*Degradation of vocal behavior*. Failed spontaneous interventions, interventions that progressively become weaker, abandoning of turn.
WHI	WHI = {S5}	*Whispering*. Talking in a low voice, with the intention of being heard by only a few people, establishing complicity. It leads to confused murmuring.
TO	TO = {TO}	*Touching*. Intentional physical contact with another person.
NOI	NOI = {MO S2 S3}	*Noise*. Noise or noises produced by a person, through their body (e.g., sneezing, burping, clapping), interaction with an object (e.g., chair, table, wall), or movement.
S1	S1	*Surrounding noise*. Sounds from outside the therapy room that are loud enough to be clearly heard.
Q	Q = {Q}	*Silence*. No words. Indicates no behavior.

It should be noted that some dimensions gave rise to a single category, but given their conceptual relevance, we considered it important to include them as dimensions in the instrument. The dimensions and categories are shown in Table 2.

### Procedure

The parents of the six adolescents were notified that their children had been proposed for group therapy after a diagnostic evaluation period. In addition, they all agreed to participate in a parallel group led by another therapist.

All the sessions were video-recorded and transcribed in full. Thirty sessions were held but due to technical difficulties with the recording, six were discarded because of poor audio. Therefore, 24 sessions were included in the final analysis. Each of the sessions lasted an hour. The sessions were grouped into four periods spanning an 8-month period.

#### Data quality control analysis: inter-observer agreement

For the data quality control analysis, two observers analyzed and coded four of the therapy sessions. They had been previously trained using the approach described by Anguera (2003). Agreement was assessed quantitatively using Cohen's kappa statistic (Cohen, 1960, 1968) in GSEQ5 (version 5.1) following the recommendations of Bakeman and Quera (1995a,b, 2001, 2011). According to the criteria of Landis and Koch (1977), the level of agreement was “almost perfect”, with kappa values ranging between 0.86 and 0.93 for all the sessions.

#### Data analysis

Polar coordinate analysis was used to analyze DYN and MNT interventions in accordance with the study objective. Polar coordinate analysis is a commonly used quantitative analytical method in observational methodology that identifies the statistical relationship between a behavior of interest (referred to in polar coordinate analysis as the *focal behavior*) and other behaviors (referred to as *conditional behaviors*). Associations between pairs of behaviors are represented graphically by vectors. Polar coordinate analysis requires a prior stage consisting of lag sequential analysis (Bakeman, 1978, 1991), a technique used to reveal behavioral patterns based on occurrence of behaviors after (prospective) or before (retrospective) a given behavior (as the focal behavior is known in lag sequential analysis). The technique is based on calculating conditional and unconditional probabilities (based, respectively, on matched frequencies and simple frequencies) for each of the time lags considered, which may be positive or negative.

Lag sequential analysis produces large volumes of data, which are subsequently reduced through a powerful data reduction algorithm based on the Z_sum_ = $\frac{\Sigma z}{\sqrt{n}}$ parameter proposed by Cochran (1954), where *z* is the standard value corresponding to each lag for each of the conditional behaviors (known as *target* behaviors) and *n* is the number of lags considered. The Z_sum_ is calculated for each target behavior for both positive lags (prospective Z_sum_) and negative lags (retrospective Z_sum_). The technique thus yields a statistical relationship between the given behavior and each of the target behaviors, which is reflected by a prospective and a retrospective Z_sum_ value, as proposed by Sackett (1980, 1987). To optimize the procedure, Anguera (1997) proposed a modification to the original technique (1980, 1987) based on the concept of genuine retrospectivity. This modified technique has been used on multiple occasions in the past two decades and was employed in the current study.

Polar coordinate analysis integrates the prospective and retrospective perspectives with the help of a vectorial map that contains four quadrants in which the prospective and retrospective Z_sum_ values are plotted along the X and Y axis, respectively. Each target behavior analysis thus can be located in one of the four quadrants depending on the combination of negative/positive signs (Table 3).

**Table 3:** Polar coordinate analysis results corresponding to interventions by the therapist (T) as the focal behavior and interventions by the participants (G D JM F L M), interventions by the co-therapist (CT), and silence as conditional behaviors.

**Category**	**Quadrant**	**Prospective perspective**	**Retrospective perspective**	**Ratio**	**Radius**	**Angle**
**BLOCK 1**						
CT	I	1.96	3.64	0.88	4.13 (^*^)	61.78
G	III	−3.59	−5.52	−0.84	6.58 (^*^)	236.98
D	I	4.03	2.57	0.54	4.78 (^*^)	32.52
JM	I	1.27	2.86	0.91	3.13 (^*^)	66.01
F	I	3.69	6.02	0.85	7.06 (^*^)	58.45
L	III	−4.54	−5.7	−0.78	7.28 (^*^)	231.49
M	III	−4.51	−2.93	−0.54	5.38 (^*^)	212.99
Q	I	3.01	3.48	0.76	4.61 (^*^)	49.13
**BLOCK 2**						
CT	I	4.81	4	0.64	6.26 (^*^)	39.74
G	III	−7.33	−5.58	−0.61	9.21 (^*^)	217.31
D	III	−6.62	−5.93	−0.67	8.89 (^*^)	221.85
JM	IV	0.07	−0.21	−0.95	0.22	288.21
F	I	3.8	0.86	0.22	3.89 (^*^)	12.74
L	IV	0.81	−1.06	−0.8	1.34	307.19
M	I	5.17	5.39	0.72	7.47 (^*^)	46.16
Q	I	5.15	5.4	0.72	7.46 (^*^)	46.39
**BLOCK 3**						
CT	I	8.63	7.43	0.65	11.38 (^*^)	40.71
G	III	−16.95	−16.15	−0.69	23.41 (^*^)	223.62
D	III	−19.21	−15.73	−0.63	24.83 (^*^)	219.32
JM	I	7.42	8.75	0.76	11.48 (^*^)	49.71
F	I	13.93	11.89	0.65	18.32 (^*^)	40.46
L	III	−2.08	−5.61	−0.94	5.99 (^*^)	249.64
M	III	−1.83	−3.04	−0.86	3.55 (^*^)	239.03
Q	I	6.48	6.39	0.7	9.1 (^*^)	44.59
**BLOCK 4**						
CT	I	8.36	7.87	0.69	11.48 (^*^)	43.26
G	III	−5.44	−4.19	−0.61	6.86 (^*^)	217.62
D	III	−11.09	−9.16	−0.64	14.38 (^*^)	219.56
JM	III	−2.66	−2.93	−0.74	3.96 (^*^)	227.78
F	I	6.57	3.78	0.5	7.58 (^*^)	29.92
L	III	−11.6	−14.96	−0.79	18.93 (^*^)	232.22
M	III	−5.97	−7.98	−0.8	9.97 (^*^)	233.19
Q	I	3.98	10.16	0.93	10.91 (^*^)	68.59

* *Significant relationships (p < 0.05) between the focal behavior and conditional behaviors*.

Polar coordinate analysis uses the prospective and retrospective Z_sum_ values for each conditional behavior to calculate the length and angle of the corresponding vector, thus allowing these to be graphically represented. The length of the vector is $\surd \left(Z_{sum}^{2}Prospective+Z_{sum}^{2}Retrospective\right)$, and is considered to be statistically significant (*p* < 0.05) when it exceeds 1.96 The angle of the vector is calculated as follows: φ = arc sen $\frac{Z_{sum}Retrospective}{Length}$ and it is then adjusted according to the quadrant in which it is located: quadrant I (0 < φ <90) = φ; quadrant II (90 < φ <180) = 180 − φ; quadrant III (180 < φ < 270) = 180 + φ; quadrant IV (270° < φ < 360°) = 360° − φ.

**Figure 1:**
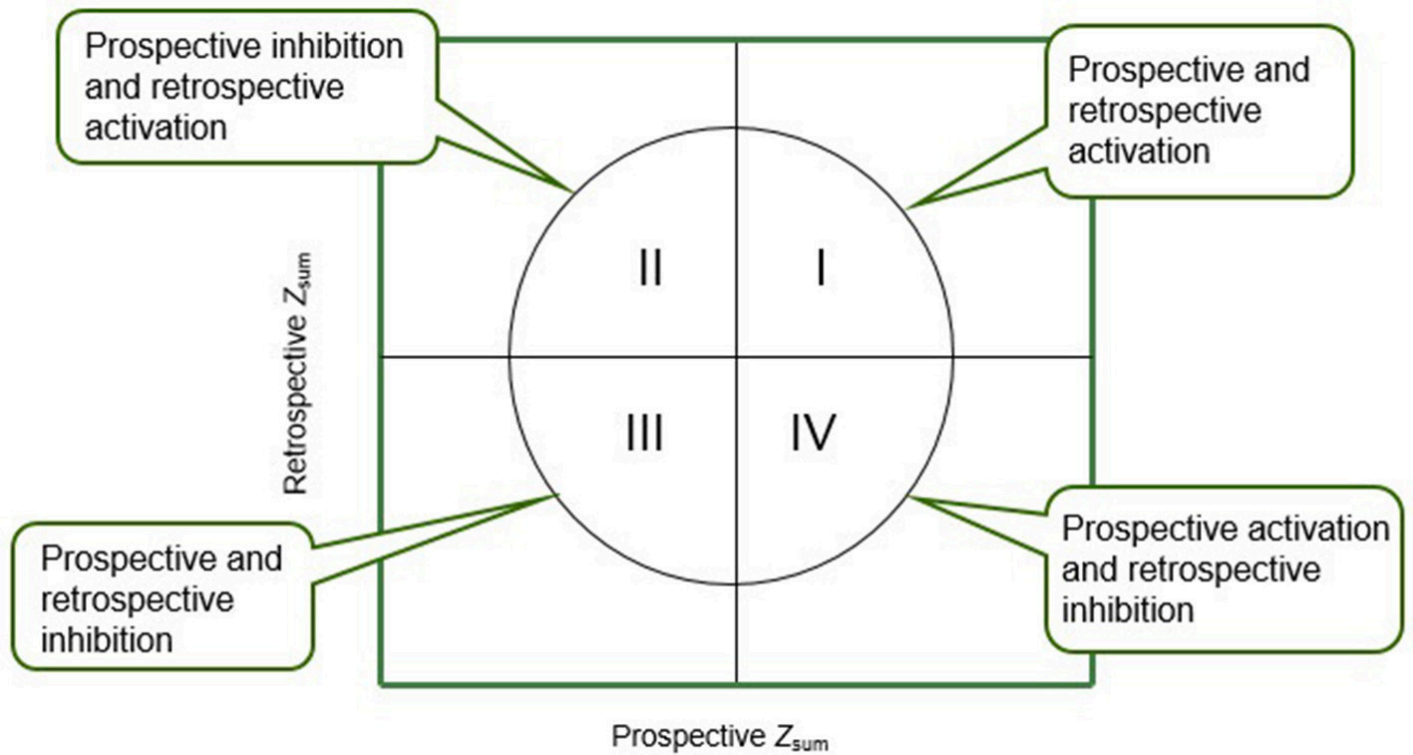
Characteristics of the quadrants in which the vectors are located according to the activation (+) or inhibition (–) sign carried by the Prospective and Retrospective Zsum values.

The meanings of the different quadrants are shown in Figure 1.

Quadrants I and III are symmetrical in terms of the relationship they depict between the focal behavior and the different conditional behaviors they contain. Quadrant I (++) indicates mutual activation while quadrant III (−) indicates mutual inhibition. Quadrants II and IV, in turn, depict asymmetrical relationships. Quadrant II (−+) indicates that the focal behavior inhibits but at the same time is activated by the conditional behaviors, while quadrant IV (+−) indicates the opposite (i.e., the focal behavior activates and is inhibited by the corresponding conditional behaviors).

The polar coordinate analysis for this study was performed in HOISAN v. 1.6.3.2 (Hernández-Mendo et al., 2012), which contains all the necessary modules and also produces partial results for adjusted residuals and *z* values in addition to analytical parameters and polar coordinate maps. The analysis was conducted by exporting the data file from GSEQ5 to HOISAN.

Polar coordinate analysis has been used in certain areas of clinical psychology, such as groups of children with autistic siblings (Venturella, 2016). It has also been widely applied in sports (Perea et al., 2012; Robles-Prieto et al., 2014; Echeazarra et al., 2015; López-López et al., 2015; Morillo-Baro et al., 2015; Sousa et al., 2015; Castañer et al., 2016, 2017; Aragón et al., 2017) and school settings (Herrero Nivela, 2000; Anguera et al., 2003; López et al., 2016; Santoyo et al., 2017). As a final note of interest, when Sackett (1980) first presented polar coordinate analysis, he used it to study turn-taking in conversation.

## Results and discussion

In the sections below, we describe the relationships detected between interventions by the therapist and the group participants using polar coordinate analysis.

### Relationships between turn-taking by the therapist, turn-taking by the participants and the co-therapist, and silence

The focal behavior was intervention by the therapist (T) and the conditional behaviors were interventions by the participants (G, D, JM, F L, and M), interventions by the co-therapist (coT), and silence (Q) in the four blocks of sessions spanning 8 weeks.

As shown in Table 3, the majority of results were significant.

**Figure 2:**
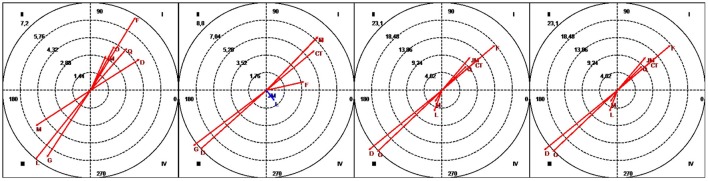
Vectors corresponding to interventions by the therapist (T) as the focal behavior and interventions by the participants (G, D, JM, F L, and M), interventions by the co-therapist (coT), and silence (Q) as conditional behaviors. Session blocks 1-2-3-4 (from left to right).

The graphs in Figure 2 show the vectors representing turn-taking by the participants and the co-therapist and silence. In the case of the adolescents, some of the vectors are located in the mutual inhibition quadrant (quadrant III) while others are located in the mutual activation quadrant (quadrant I). On analyzing the four blocks of sessions grouped by time, it can clearly be seen that the turn-taking behavior by D, L, and M changed over the course of therapy, that of the co-therapist and silence remained stable.

### Relationship between the therapist and DYN and MNT interventions

Again, the focal behavior was intervention by the therapist (T) and the conditional behaviors were the DYN categories FF, FO, RP, RT, QA, QC, and QV and the MNT category.

The majority of results in this case were also significant (Table 4).

**Table 4:** Polar coordinate analysis results with interventions by the therapist (T) as the focal behavior and DYN categories (broken down) and MNT as conditional behaviors.

**Category**	**Quadrant**	**Prospective perspective**	**Retrospective perspective**	**Ratio**	**Radius**	**Angle**
**BLOCK 1**						
QA	IV	2.36	−3.87	−0.85	4.54 (^*^)	301.4
QC	III	−4.61	−4.61	−0.71	6.52 (^*^)	224.98
FF	II	−0.42	1.94	0.98	1.99 (^*^)	102.32
FO	IV	0.11	−1.99	−1	2 (^*^)	273.02
RP	I	1.28	5.16	0.97	5.32 (^*^)	76.07
QV	III	−1.53	−1.59	−0.72	2.2 (^*^)	226.01
RT	II	−0.07	0.31	0.98	0.32	102.7
MNT	III	−3.91	−1.89	−0.44	4.34 (^*^)	205.83
**BLOCK 2**						
QA	IV	4.9	−1.81	−0.35	5.22 (^*^)	339.72
QC	III	−3.54	−2.49	−0.58	4.33 (^*^)	215.2
FF	I	1.36	3.06	0.91	3.35 (^*^)	66.01
FO	I	0.74	2.15	0.95	2.28 (^*^)	71.07
RP	I	4.83	6.78	0.81	8.33 (^*^)	54.5
QV	I	1.46	1.07	0.59	1.81	36.25
RT	II	−2.56	0.47	0.18	2.61 (^*^)	169.63
MNT	II	−2.88	1.96	0.56	3.48 (^*^)	145.78
**BLOCK 3**						
QA	L	14.08	6.32	0.41	15.43 (^*^)	24.17
QC	III	−1.11	−3.01	−0.94	3.21 (^*^)	249.85
FF	I	3.49	5.31	0.84	6.35 (^*^)	56.66
FO	I	3.61	4.11	0.75	5.47 (^*^)	48.65
RP	I	3.72	4.87	0.79	6.13 (^*^)	52.64
QV	I	5.39	3.69	0.56	6.54 (^*^)	34.38
RT	II	−2.11	3.14	0.83	3.78 (^*^)	123.86
MNT	II	−0.32	5.49	1	5.5 (^*^)	93.33
**BLOCK 4**						
QA	I	10.12	4.3	0.39	10.99 (^*^)	23.01
QC	IV	0.38	−1.51	−0.97	1.56	284.25
FF	I	4.96	6.75	0.81	8.38 (^*^)	53.72
FO	I	4.7	3.78	0.63	6.03 (^*^)	38.83
RP	I	4.59	5.54	0.77	7.2 (^*^)	50.39
QV	I	4	1.46	0.34	4.26 (^*^)	20.04
RT	II	−1	2.74	0.94	2.91 (^*^)	110.1
MNT	I	3.86	9.43	0.93	10.19 (^*^)	67.76

* *Significant relationships (p < 0.05) between the focal behavior and conditional behaviors*.

**Figure 3:**
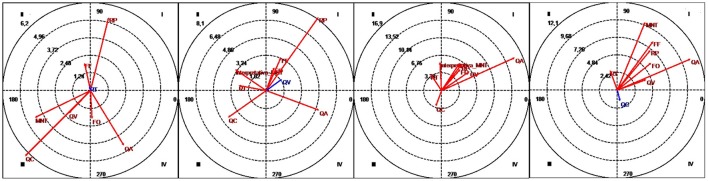
Vectors corresponding to interventions by the therapist (T) as the focal behavior and conversationfacilitating DYN categories (FF, FO, RP, RT, QA, QC, QV) and the mentalizing or reflective function MNT category as conditional behaviors. Session blocks 1-2-3-4 (from left to right).

The graphs in Figure 3 show the vectors for the different relationships distributed among the four quadrants. On examining the figures by blocks of time, it can be seen that the vectors tend to form clusters, with the majority located in the mutual activation quadrant (quadrant I) by the end of the therapy. Note that the length of the radius for repetition (RP) and the quadrant in which it was located (quadrant I) remained stable over the four periods.

Below we discuss the significance of the relationships detected by polar coordinate analysis in five sections. We also illustrate our findings with clinical vignettes containing coded transcripts of the interventions.

### Turn-taking by the therapist and the adolescents

All the significant results are located in two opposing quadrants, indicating two clearly differentiated types of relationship: mutual activation and mutual inhibition. The therapist always facilitates intervention by Fred, the participant with the greatest difficulty relating to others, and in the early phases of therapy, she also encourages interaction from Danny, John M, and Meg. Her interventions never activate those of the two impulsive participants, Gabriel and Lucy. This does not mean that she excludes these participants, simply that they intervene on their own initiative. The changes detected in Danny, John M, and Meg are an indication of the progress they make over the therapy. Block 1 is characterized by radial communication between the therapist and all the participants. Vignette 1 shows an example of an interaction between the therapist and Danny (Table 5).

**Table 5:** Clinical vignette 1.

Vignette 1 (Block 1). Danny has been on a trip to a museum with his school.
T – It's a different museum, right? [QA]
D – Yes, it was an industry. [RA]
T – It was an industry; is it located in an old factory? [RP] [QA]
D – Yes, in a factory, they used an industry from the 1960s. [RA]
T – Hmmm…And you said that you had to do an assignment? [FF] [PA]
D – They gave us a sheet of paper and we had to fill it in. [RA]
T – With the things you were seeing and the explanations they were giving you? [QA]
D – Yes. [RB]

However, not all interactions are the same. Gabriel and Lucy, for example, spontaneously take turns in these early sessions (Table 6).

**Table 6:** Clinical vignette 2.

Vignette 2 (Block 1). The topic of conversation is about getting down to studying and passing and failing subjects
G – Yes, at the beginning you see it as far off, Well…that's what I think, and you do nothing. [RA]
T – Hmmm. [FF]
G – But then, when you see that you are getting bad marks, and that if you don't get your act together, well, they will fail you, then you study. [EC]
T – Is that the same with all of you? [QA]
L – For me it's the opposite. [RA]
T – Aha. [FF]
L – In the first, in the first term, well that was it, I had to study, and because I spent the summer studying…, I mean, I don't care, the truth is that it doesn't matter if it's at the beginning of the year or at the end [EC]
G – That's the bad thing, like she says, yes, because if you have to study in September, yuck! In my school, they do courses in July, right there, and I spend a month at school. They give you minimum goals and at the end of the course, they test you, you can do at least three…[EC]
L – Yeah, well imagine if you've got seven subjects left for the summer, for September. [EC]

Lucy raises conflicts about herself that interest everyone (Table 7).

**Table 7:** Clinical vignette 3.

Vignette 3 (Block 1).
Lucy has just explained that she has been to different schools:
T – And now, how are you? (current school) [QA]
L – Fine, but I don't like it, I don't like any of the girls in my class. [RA]
T – What do you mean? What don't you like about the girls in your class? [QC] [QA]
L – That they're always saying I'm very childish because I don't wear make-up or show my thighs, I don't like that! [RA]
T – Hmmm [FF]
L – And they say I'm very childish because I'm 15 but I don't like wearing make-up or going off into corners kissng guys. I'm not into that, but that's what they appear to do. [EC]
T – Hmmm. [FF]
L – And when they ask me if I'm coming with them, I don't go. I'm not into that [EC]
T – Hmmm. What do the rest of you think about what Lucy is saying? [FF] [QA]
M – Good. [RB]
T – Good. What do you mean? [RP] [QC]
M – That…She will end up better than them, they're the ones going astray. [RA]

John M is a reserved person with anxiety problems. He has difficulty intervening and when he does, he often mumbles, says very little, and adheres to what has just been said (Table 8).

**Table 8:** Clinical vignette 4.

Vignette 4 (Block 1).
The topic of conversation is about marks and exams. They have all explained how they are assessed. John M says nothing until the therapist asks him directly.
T – And what about you, John M? How are you assessed? [QA]
JM – Like her. [RA]
I suppose you're referring to Lucy, who has just spoken.
T – Exactly exactly like her? [QA]
JM (in a low voice)- Yes [RB]

Haen and Weil (2010) have highlighted the difficulties that adolescents have engaging during this initial stage of therapy. In our study, as the therapy progresses, the adolescents start to communicate much more naturally and spontaneously and bring up issues that concern them, such as going out, the end of the school year, and their expectations for the coming year. Vignette 5, which contains an excerpt from this last block, shows how Danny, John M, Lucy, and Meg chat freely amongst themselves, without encouragement from the therapist. Amidst jokes, exclamations, gestures, and laughter, they talk about meeting outside the group and about their fears of traveling alone on the train or underground for the first time (Table 9).

**Table 9:** Clinical vignette 5.

Vignette 5 (Block 4).
Lucy is explaining that she's going to be in a play in a village near the Mental Health Center. Meg asks her directly:
M (addressing L) – And you don't feel embarrassed? [QA]
L – Yes, and they say that they're going to throw eggs at us. [RA]
D – Jeez. [EE]
JM – Count me in. [EO]
D – You know what I mean, yahoo! One by one! (gestures of throwing eggs) [EO] [EE] [EO]
JM – Haha. [R]
L – I hope they're joking, because if not, they'll get in trouble. [CFR]
M (addressing L) – Can you get there by train? [QA]
L – Yes. [RB]
M (in a low voice) – Darn. [EE]
L – If you can get there by train? [RT]
D – I'll bring some hens, hahaha. [EO] [R]
JM – Let's go, yay! [EO] [EE]
M – You get there by train? [PV]
D – Yes! [RB]
L – Or you can go by car or…[EC]
D – There are tracks and a station, hahaha. [EO] [R]
M – Bah! I'm not going by train. [EE] [EC]
JM – Hee hee. [R]
JM – Hee hee hee. How are you going to go, on foot? Haha. [R] [EO] [R]
JM – Haha. [R]
M – Haha. No. [R] [RB]
M – No, because of what happens to her with the underground (referring to being afraid to ride alone) [EXP]
JM and D in unison – The same things happens to you with the train. [CFR]
M – No, because the first time I go on a train alone, well …[DEF]
D – You'll get lost…[CFR]
M – No…[DEF]

### Turn-taking by the therapist and the co-therapist

The co-therapist and the therapist was mutually activated (quadrant 1). The co-therapist's interventions reflect her role of interfering as little as possible in the group dynamics. They complement those of the main therapist. Together, they form a team and create and maintain a safe environment (Shechtman 2007; Torras de Beà, 2013; Malekoff, 2014).

### The therapist and silence

The therapist generates silence but also breaks it (quadrant 1).

The examples below show how the adolescents fall silent when faced with difficult issues, such as verbalizing why they are in the group or talking about their relationship with their parents or their concerns about sexuality (Tables 10–12).

**Table 10:** Clinical vignette 6.

Vignette 6 (block 2). The therapist challenges the participants with questions, she takes them to a level of mentalization that they are not ready for yet and they become inhibited.
T – Why are we coming to the group? And why? We are all coming for something, aren't we? [MNT]
Silence. [Q]
T – Why do you think you are coming? How are we are trying to help you here? [MNT]
Silence. [Q]
T – Maybe we have to go over this again…[EXP]

**Table 11:** Clinical vignette 7.

Vignette 7 (block 3). At another moment, silence allows the adolescents to express themselves with sincerity:
T – How would you like your parents to treat you? What do you expect? [MNT]
Silence. [Q]g
D – Them not to use such tough punishments [EXP]
T – Not to use such tough punishments [EXP]
G – They always use the worst possible punishments [EXP]
T – The worst? What does that mean, what you like most? [QV] [MNT]
G – Yes, they punish you with the things you like most. [EC]
T – And what happens then? How do you feel? [MNT]
G (in a very low voice) – Crap…[EXP]
Silence. [Q]
T – How do you all feel? Do you get discouraged? Do you feel that they are disheartening you? [MNT]
Silence. [Q]

**Table 12:** Clinical vignette 8.

Vignette 8 (Block 3)
This silence expresses the difficulty talking about sexuality.
T – Maybe you talk about condoms at school, do you? Amongst ourselves too, right? [QA]
Silence. [Q]
T – No? [PV]
JM – Haha. [R]
G – Haha. [R]
T – Jokingly, jokingly, it makes you laugh. I think that it is, that it's something that's talked about at school, about their use, right? [MNT] [QA]
Silence. [Q]
T – You're all a little quiet, aren't you? Eh? What do you think about condoms? Do you know anything? Do you talk about them with each? [MNT] [A]
Pause. [Q]
T – Before you were talking about AIDS, somebody said this word like with a lot of disgust, about the risk of infection …[MNT]
G burps and covers his mouth, mumbles something to D that I don't understand. [NOI] [S4]
D – Brrr. [EE]
G – But blood doesn't have to come out to get an infection. [EXP]

### The therapist and DYN interventions

The different strategies for facilitating conversation (FF, FO, RP, RT, QA, QC, and QV) showed varying patterns of change over the course of therapy but converged at the end.

Repetition (RP) was the most powerful strategy, as it activated conversation from the start of the therapy program. The next most powerful strategies were phatic function (FF) and greetings (FO). The transcripts of the sessions show that in the early sessions, it was the therapist who verbally greeted the adolescents (by saying hello and goodbye). However, few of them responded and the others returned the greeting or made a non-verbal gesture. This behavior changes after the first block, indicating an increase in reciprocity between the therapist and the participants.

The appearance of QA (questions directed at others) in the second half of the therapy is, in our opinion, a highly significant indicator of the group process. It tells us that the communication is no longer radial and that the adolescents have achieved one of the most important benefits of group therapy, which is showing interest in others (Yalom, 2005) in the presence of the therapist (Torras de Beà, 2013).

It is also interesting to see how QV (repetition of a previous utterance in the form of a question) changes from being mutually inhibitory to being mutually activating. We think that this strategy initially surprised the adolescents but was then gradually adopted by them. The same was not observed for QC (clarifying questions), which were used only by the therapist when the adolescents were “doing their own thing” and she was “excluded” from the group. Examples of what she said were: “I'm not quite following you now…maybe I'm being a bit dense, can you help me understand what's going on?” This strategy is similar to the attitude of respectful curiosity shown by therapists in the Adolescent Mentalization-Based Integrative Treatment (AMBIT) approach (mentalizing stance), which is designed to help put a halt to non-mentalization mental states (Benvington et al., 2012; Dangerfield, 2016).

Bringing back a central topic of conversation (RT) and suggesting looking at this in greater depth was only done by the therapist.

At the end of therapy, all the categories in the DYN dimension except RT are located in the mutually activating quadrant. This supports the idea that the communication strategies used by the therapist were adopted by the participants, enabling them to talk more autonomously and facilitating their personal growth (Yalom, 2005; Torras de Beà, 2013).

### The therapist and MNT interventions

The changes observed in the MNT category, which corresponds to interventions aimed at improving the adolescents' mentalization abilities, also reflect interesting aspects of the group process. The MNT category changed from inhibitory (quadrant III) to partially inhibitory (quadrant II) and finally to mutually activating (quadrant I). The changes also show that the therapist's role changed over time, as mentalization strategies were only used by her. We can deduce that the participants gradually overcame their early inhibitions and dependence and acquired more sophisticated mentalizing abilities, helping them to become more aware of themselves and of others. This result is consistent with the concept known as the interpretative function of the therapist within the theories of Foulkes (1986) and Torras de Beà (2013).

## Conclusions

Polar coordinate analysis provides a new approach for gaining insights into dialogue in group pyschotherapy. The results show that the technique provides a novel means of analyzing the role of the therapist and describing her conversational style. The therapist proved to be an expert in creating a communicative environment that allowed the adolescents to grow. She employed four core strategies: (1) she did not facilitate communication equally for all participants, (2) she encouraged turn-taking by the more inhibited members of the group, (3) she stimulated conversation from the early stages of therapy, and (4) she promoted mentalization toward the end of therapy.

We were particularly pleased to see that the use of repetition (RP) facilitated communication flows from the beginning. The positive results indicate that rather than simply acting as an echo or a loudspeaker, this strategy produces a mirroring effect similar to that described in the social biofeedback theory of parental affect-mirring (Gergely and Watson, 1996), in which the person talking, apart from being listened to, is brought into a mirror-like interaction. This regulatory effect is a prerequisite for the mentalization process that facilitates the development of the self (Fonagy et al., 2002).

Observational methodology and polar coordinate analysis could prove to be of great value for detecting changes in psychotherapy models based on spoken conversation.

## Author contributions

EA developed the project. MA performed the method section and polar coordinate analysis. Both authors have participated in the writing of the article.

### Conflict of interest statement

The authors declare that the research was conducted in the absence of any commercial or financial relationships that could be construed as a potential conflict of interest.
